# Supplementary material for: Controlled Release Mechanism of Vancomycin from Double-Layer Poly-L-Lactic Acid-Coated Implants for Prevention of Bacterial Infection
Source: Polymers (Basel). 2022 Aug 26;14(17):3493. doi: 10.3390/polym14173493 (PMC9460839; doi:10.3390/polym14173493)
Supplement: Supplementary file 1 [file polymers-14-03493-s001.zip › polymers-1858945-supplementary.pdf]

Supplementary Materials

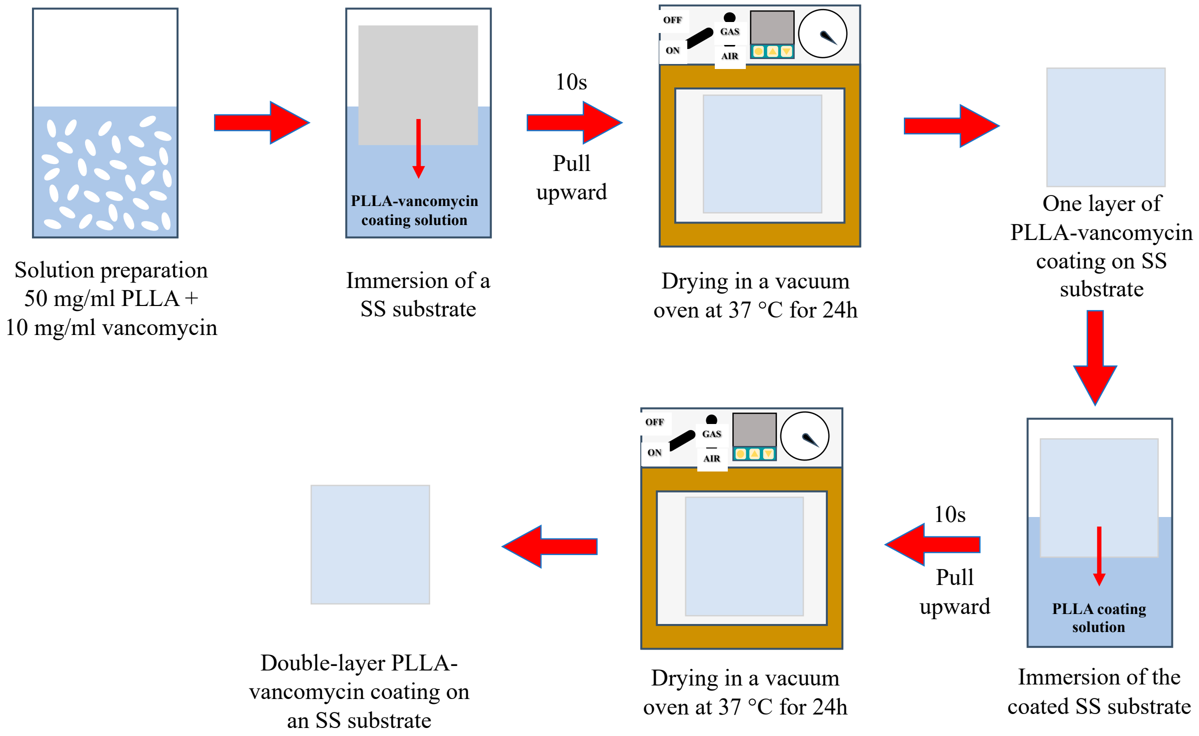

Figure S1. Schematic diagram of the preparation procedure of the double-layer PLLA-vancomycin coating on a stainless steel substrate. Note that SS represents a stainless steel substrate.

Table S1. Preparation and composition of simulated body fluid (SBF).

All reagents (Sigma-Aldrich, Singapore) in the table below were added to 500 mL of distilled water under constant stirring until the solution was homogeneous. The temperature and pH of the buffer were adjusted to 36.5 °C and 7.40, respectively. The total volume of the buffer was finally adjusted to one liter. The buffer was stored at 4 °C until use.

Reagents for SBF preparation:

Order	Reagent	Amount
1	$NaCl$	7.996 g
2	$NaHCO_3$	0.350 g
3	KCl	0.224 g
4	$K_2HPO_4 \cdot 3H_2O$	0.228 g
5	$MgCl_2 \cdot 6H_2O$	0.305 g
6	1M–HCl	40 mL
7	$CaCl_2$	0.278 g
8	Na_2SO_4	0.071 g
9	$(CH_2OH)_3CNH_2$	6.057 g

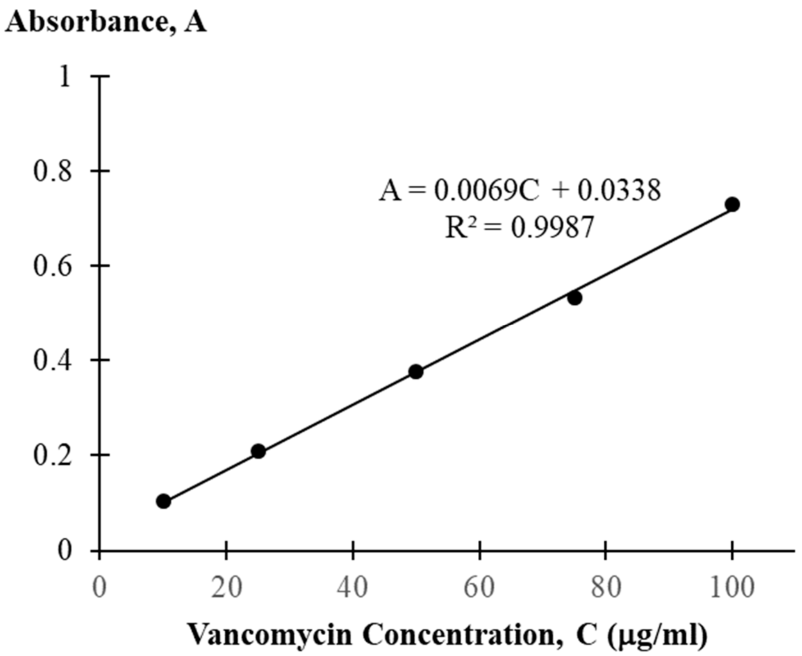

Figure S2. Calibration curve of vancomycin in SBF for colorimetric quantitation.
